# Hepatoid Adenocarcinoma Presenting as Pancreatitis

**DOI:** 10.14309/crj.0000000000000381

**Published:** 2020-05-07

**Authors:** Rahul Chaudhari, Katherine Murphy, Stephen Schwartz, Jigisha Chaudhari, Immanuel Ho, Frederick Nunes

**Affiliations:** 1 Department of Medicine, Pennsylvania Hospital of the University of Pennsylvania Health System, Philadelphia, PA; 2 Department of Pathology, Pennsylvania Hospital of the University of Pennsylvania Health System, Philadelphia, PA

## Abstract

Hepatoid adenocarcinoma (HAC) is an uncommon and aggressive type of adenocarcinoma, typically affecting the middle-aged and elderly. The morphological features of the HAC resemble hepatocellular carcinoma. Presenting symptoms may include upper abdominal pain, hematemesis, back pain, and palpable abdominal mass. HAC has no proven therapy, and the prognosis is extremely poor. Early surgical removal with chemotherapy remains the standard of care. We describe one of the youngest patients in the literature with HAC who presented with acute pancreatitis. The diagnostic workup was confused by diffuse lymphadenopathy and elevated β-human chorionic gonadotropin making lymphoma and germ cell tumor likely possibilities until immunohistochemistry confirmed the diagnosis.

## INTRODUCTION

Hepatoid adenocarcinoma (HAC) is a rare extrahepatic subset of adenocarcinoma associated with elevated α-fetoprotein (AFP). It is often metastatic at the time of diagnosis and carries a poor prognosis overall. The morphology of HAC closely resembles that of hepatocellular carcinoma (HCC), both of which commonly produce AFP presenting a diagnostic challenge. The diagnosis is usually made by hematoxylin and eosin staining and immunohistochemical (IHC) staining. HAC typically presents in the fourth to sixth decade. Presenting symptoms include upper abdominal pain, hematemesis, back pain, weakness, and palpable abdominal mass. We present one of the youngest cases of HAC reported in the literature involving a 28-year-old man with acute pancreatitis making it a rare presenting symptom.

## CASE REPORT

A 28-year-old previously healthy white man of eastern European descent presented with a 2-week history of vomiting, anorexia, and abdominal pain. Physical examination was significant for mild tenderness in the epigastrium and left upper abdomen. Routine laboratory investigations were remarkable for abnormal liver blood tests—aspartate aminotransferase was 150 U/L, alanine aminotransferase was 387 U/L, alkaline phosphatase was 234 U/L, and total bilirubin was 2.5 mg/dL. Lipase was elevated at 1132 U/L and lactate dehydrogenase was 391 mg/dL. Abdominal ultrasound revealed lymphadenopathy posterior to the pancreatic head and dilatation of the proximal extrahepatic bile duct up to 11 mm.

**Figure 1.**
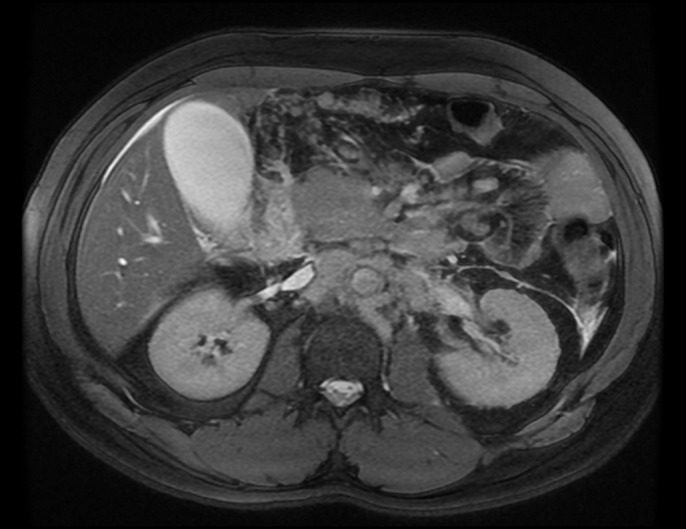
Magnetic resonance cholangiopancreatography showing diffuse lymphadenopathy with mild intrahepatic biliary ductal dilatation.

**Figure 2.**
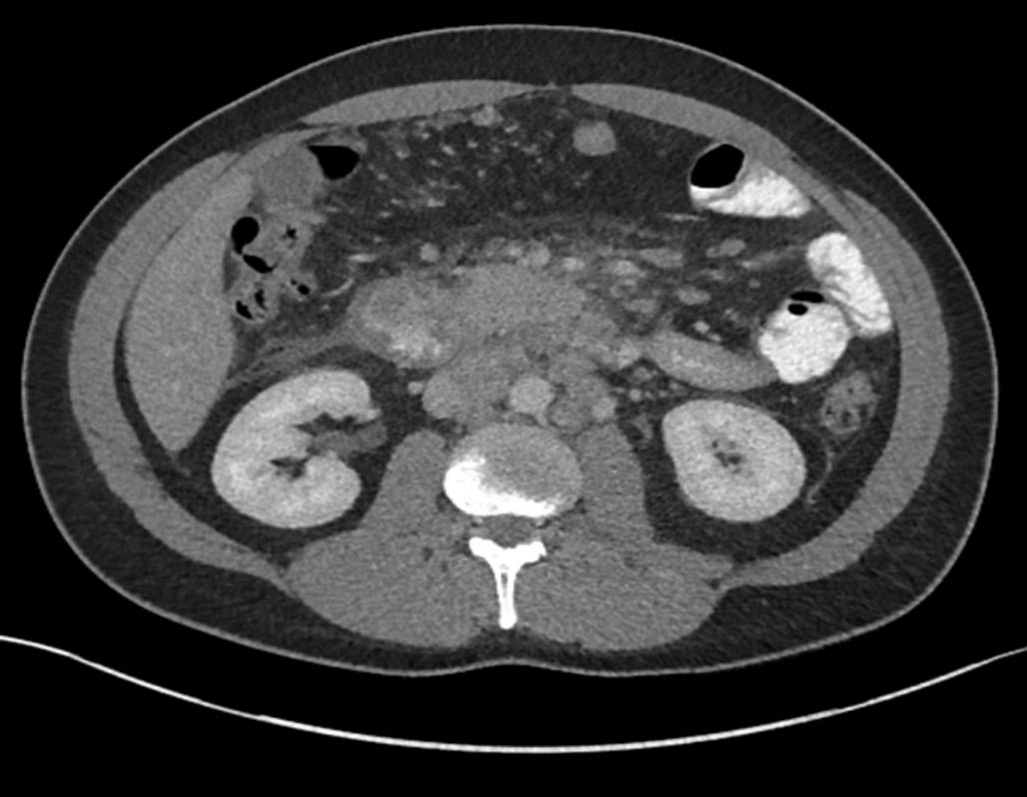
Abdominal computed tomography showing inflammation around the pancreatic head and the duodenum with retroperitoneal and peritoneal lymphadenopathy.

Magnetic resonance cholangiopancreatography showed bulky retroperitoneal, periportal, mesenteric, retrocrural, and lower thoracic lymphadenopathy and extrahepatic and intrahepatic biliary ductal dilatation with ill-defined thickening of the gastric antrum (Figure 1). Subsequent laboratory investigations revealed elevated β-human chorionic gonadotropin (β-hCG) of 872 mIU/mL and AFP of 244 IU/L. Testing for antinuclear antibody, human immunodeficiency virus, and anti-immunoglobulin G antibodies was negative. Thoracic, abdominal, and pelvic computed tomography showed acute interstitial edematous pancreatitis, mural thickening of the distal gastric body, and duodenum with diffused lymphadenopathy suggestive of lymphoma (Figure 2).

**Figure 3.**
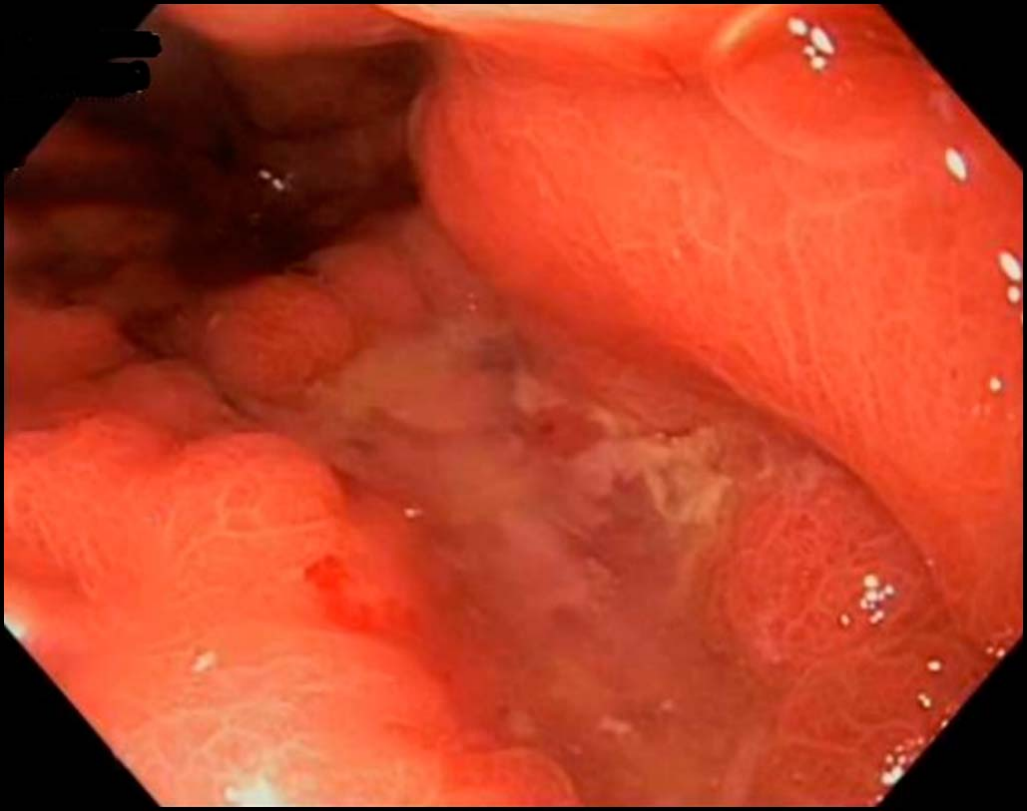
Nonbleeding cratered gastric ulcer (10 mm at the largest dimension) with no stigmata of bleeding on the lesser curvature of the stomach.

**Figure 4.**
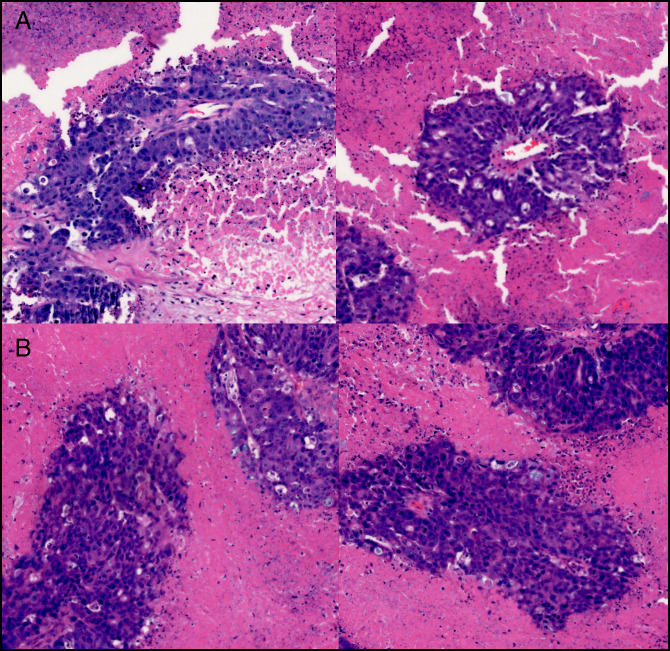
(A and B) Histology of hepatoid adenocarcinoma from gastric, duodenal, and bile duct brushing. Note the mixture of glandular cells and cells with hepatoid differentiation.

Surgical excisional biopsy of supraclavicular lymph node was performed for tissue diagnosis. Initial endoscopic retrograde cholangiopancreatography (ERCP), performed for abnormal liver function tests (total bilirubin 6.4 mg/dL on the fifth day from 2.5 mg/dL on admission) and the need for biliary decompression, showed nonbleeding cratered gastric ulceration on lesser curvature, duodenal infiltration, and biliary stricture requiring stenting with a plastic stent and obtained the biliary brushing (Figure 3). The patient then developed ascending cholangitis with rising bilirubin and underwent a repeat ERCP that upsized the stent and changed it to a metal stent, followed by a cholecystostomy for poor surgical candidacy. After initial improvement to 1.2 mg/dL, total bilirubin increased to 7.3 mg/dL requiring another ERCP that found occlusion of the newly placed stent from tumor debris and 2 other plastic stents were placed. Subsequently, the result of lymph node biopsy revealed germ cell tumor (positive for β-hCG) without lymphoproliferative changes, excluding lymphoma. A testicular ultrasound to rule out the testicular tumor was unremarkable. In the meantime, the biopsy from biliary brushing and the gastric ulcer was positive for adenocarcinoma with hepatoid features and signet ring appearance (Figure 4). While admitted, he received 2 cycles of chemotherapy including 5FU, leucovorin, oxaliplatin, and docetaxel with trastuzumab (5-fluororuraci, leucovorin, oxaliplatin, docetaxel + trastuzumab). He received a total of 9 cycles of chemotherapy with 5-fluororuraci, leucovorin, oxaliplatin, docetaxel + trastuzumab before he died due to HAC.

## DISCUSSION

HAC was first reported by Bourreille et al in 1970 after which Ishikura et al proposed the term “HAC of the stomach” because of its morphologic similarity to HCC. HAC constitutes 0.38%–1.6% of all gastric cancers, with an incidence of 0.58–0.83 cases per million inhabitants.^1–4^ Men are more commonly affected than the women.^3^ The characteristic features of HAC are hepatoid differentiation of cancer cells, the production of large amounts of AFP, and coexistence of both adenocarcinomatous and hepatocellular differentiation.^2^ The stomach is most commonly involved by HAC; however, other organs such as ovaries, lungs, urinary bladder, gallbladder, pancreas, uterus, testis, and retroperitoneum have also been reported to be involved.^5^ Patients with HAC frequently present with liver metastases.^6^

The differential diagnosis of HAC includes HCC, pancratoblastoma, acinar cell carcinoma, and neuroendocrine tumors. The clinical signs/symptoms and imaging findings are very nonspecific. Hence, the diagnosis is mainly based on the morphologic features and characteristic IHC features, irrespective of the serum AFP levels or tissue AFP staining by IHC.^7^ Imaging might show extensively thickened gastric walls (as in our case) and sometimes a polypoid mass.^8,9^ Although most patients with HAC (70%–80%) will have elevated serum AFP levels ranging 1.0 to 700,000 ng/mL, not all HAC produce AFP and almost 46% of patients with HAC do not produce AFP.^1,^2,9 Our patient had a relatively low level of AFP compared with other AFP positive HAC cases.^3,10,11^ Ectopic β-hCG production is common in epithelial tumors from ovaries, cervix, lung, colon, and mainly the stomach and might suggest an aggressive nature of the tumor.^12^

Morphologically, HAC cells are composed of large eosinophilic or clear cells in a sheet-like or trabecular pattern with sinusoidal vascular channels.^13^ At the molecular level, p53 mutation is a common finding.^14,15^ Similar to HCC, cancer cells from HAC are immunoreactive with AFP, polyclonal CEA, CK8, and CK18 and produces AFP and β-hCG.^6^ Not any single IHC stain can differentiate HAC from HCC, but a panel of IHC stains with detailed history and endoscopic and imaging findings are essential for definitive diagnosis of HAC. Typical IHC profile promising in the differential diagnosis of HAC include anti-sal-like protein 4 (SALL-4) glypican-3, α1-AT, α1-antichemotrypsin, CD10, CDX-2, MUC1, and PLUNC.^5,16^ Moreover, AE1/AE3, CK18, and CK19 are usually positive among epithelial markers in HAC.^5^ SALL-4 expression (as SALL-4 is typically absent in normal liver tissue and HCC) and the absence of expression of bile salt export pump and multidrug-resistance protein 3—2 transporters specific to hepatocytes—are helpful in distinguishing HAC from HCC.^17,18^

The rarity of HAC precludes the establishment of standardized treatment guidelines, and most data guiding the management remain anecdotal. Surgical removal of the tumor, wherever possible, remains the cornerstone of the treatment. However, surgery is not always possible because of the aggressive nature of the tumor and rapid metastasis. Adjuvant chemotherapy has shown to improve survival.^19^ Successful treatment of HAC has been reported using cisplatin and etoposide.^20^ In metastatic disease, cisplatin-based chemotherapy appeared to be the most efficient first-line systemic treatment with almost 75% showing clinical response.^20^ Our patient died due to HAC 16 months after diagnosis. Further investigation is needed to identify the risk factors, supplement the diagnosis, and optimize the management of HAC.

## DISCLOSURES

Author contributions: R. Chaudhari, K. Murphy, S. Schwartz, and J. Chaudhari wrote the manuscript. F. Nunes and I. Ho approved the final manuscript. R. Chaudhari is the article guarantor.

Financial disclosure: None to report.

Previous presentation: This case was presented at the International Liver Cancer Association; September 20-22, 2019; Chicago, Illinois.

Informed consent was obtained for this case report.
